# Supplementary material for: Inflammatory cells dynamics control neovascularization and tissue healing after localized radiation induced injury in mice
Source: Commun Biol. 2023 May 29;6:571. doi: 10.1038/s42003-023-04939-3 (PMC10227012; doi:10.1038/s42003-023-04939-3)
Supplement: Supplementary file 2 — Supplementary Information [file 42003_2023_4939_MOESM2_ESM.pdf]

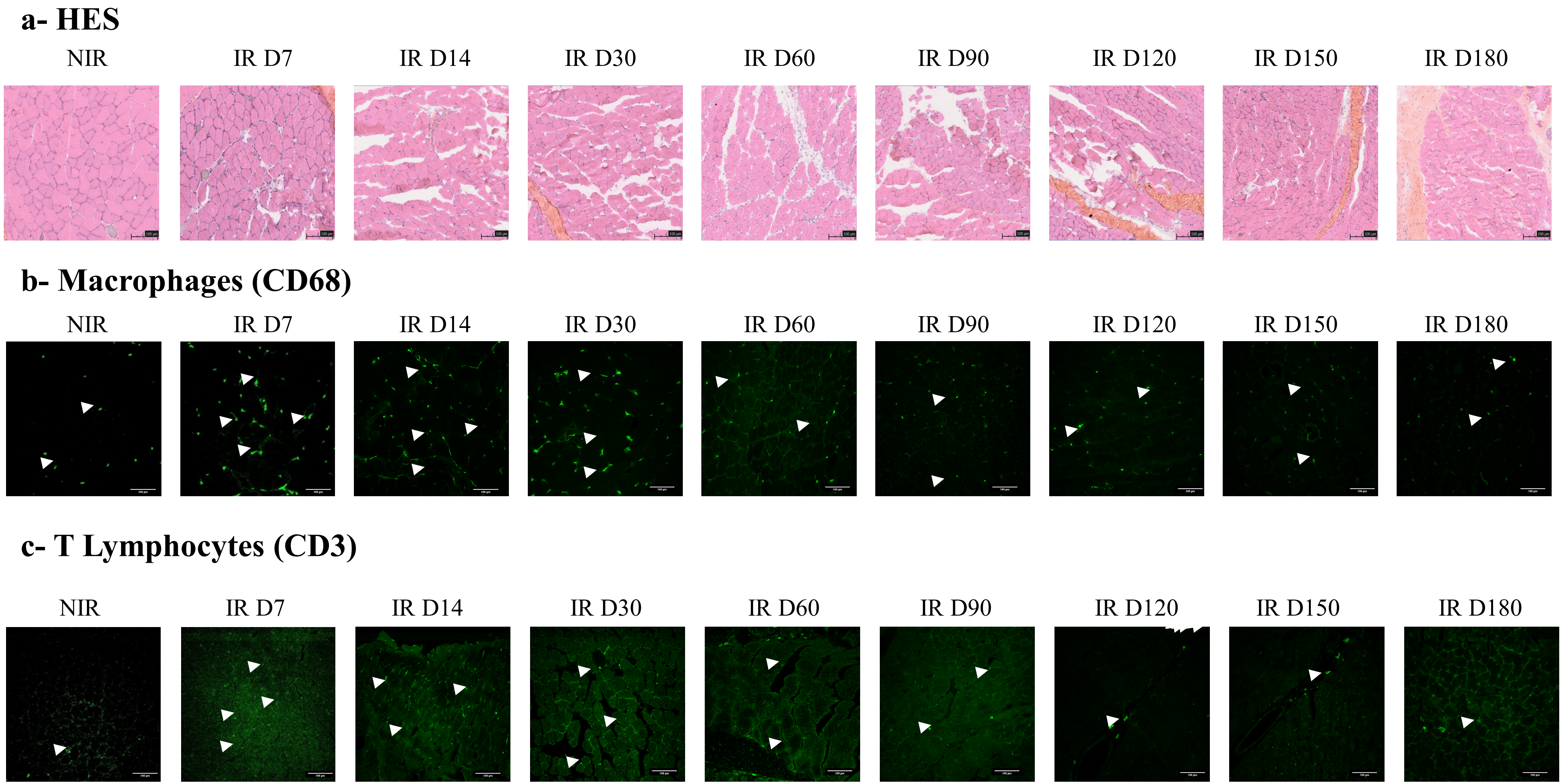

Supplementary Figure 1

Immunostaining

Representative photomicrographs of NIR and D-1, 3, 5, 7, 14, 30, 60, 90, 120, 150 and 180 post-irradiation of muscle structure stained with HES (panel a), of CD68-positive cells (panel b, indicated by arrowheads), or CD3-positive cells (panel c, indicated by arrowheads), n=6 animals/time point; bar scale: 100 µm.
