# Supplementary material for: Inflammatory cells dynamics control neovascularization and tissue healing after localized radiation induced injury in mice
Source: Commun Biol. 2023 May 29;6:571. doi: 10.1038/s42003-023-04939-3 (PMC10227012; doi:10.1038/s42003-023-04939-3)
Supplement: Supplementary file 5 — Reporting Summary [file 42003_2023_4939_MOESM5_ESM.pdf]

nature portfolio

Corresponding author(s): Céline Loinard

Last updated by author(s): 2022/07/18

Reporting Summary

Nature Portfolio wishes to improve the reproducibility of the work that we publish. This form provides structure for consistency and transparency in reporting. For further information on Nature Portfolio policies, see our Editorial Policies and the Editorial Policy Checklist.

Statistics

For all statistical analyses, confirm that the following items are present in the figure legend, table legend, main text, or Methods section.

n/a	Confirmed	
☐	☒	The exact sample size (*n*) for each experimental group/condition, given as a discrete number and unit of measurement
☐	☒	A statement on whether measurements were taken from distinct samples or whether the same sample was measured repeatedly
☐	☒	The statistical test(s) used AND whether they are one- or two-sided<br>*Only common tests should be described solely by name; describe more complex techniques in the Methods section.*
☐	☒	A description of all covariates tested
☐	☒	A description of any assumptions or corrections, such as tests of normality and adjustment for multiple comparisons
☐	☒	A full description of the statistical parameters including central tendency (e.g. means) or other basic estimates (e.g. regression coefficient) AND variation (e.g. standard deviation) or associated estimates of uncertainty (e.g. confidence intervals)
☐	☒	For null hypothesis testing, the test statistic (e.g. *F*, *t*, *r*) with confidence intervals, effect sizes, degrees of freedom and *P* value noted<br>*Give P values as exact values whenever suitable.*
☐	☒	For Bayesian analysis, information on the choice of priors and Markov chain Monte Carlo settings
☒	☐	For hierarchical and complex designs, identification of the appropriate level for tests and full reporting of outcomes
☒	☐	Estimates of effect sizes (e.g. Cohen's *d*, Pearson's *r*), indicating how they were calculated

Our web collection on statistics for biologists contains articles on many of the points above.

Software and code

Policy information about availability of computer code

Data collection	FlowJo 10, DIVA, Graphpad Prism 8, JAGS software via Markov chain Monte Carlos
Data analysis	FlowJo 10, Graphpad Prism 8, JAGS software via Markov chain Monte Carlos

For manuscripts utilizing custom algorithms or software that are central to the research but not yet described in published literature, software must be made available to editors and reviewers. We strongly encourage code deposition in a community repository (e.g. GitHub). See the Nature Portfolio guidelines for submitting code & software for further information.

Data

Policy information about availability of data

All manuscripts must include a data availability statement. This statement should provide the following information, where applicable:

- Accession codes, unique identifiers, or web links for publicly available datasets
- A description of any restrictions on data availability
- For clinical datasets or third party data, please ensure that the statement adheres to our policy

Provide your data availability statement here.

Human research participants

Policy information about studies involving human research participants and Sex and Gender in Research.

Reporting on sex and gender	*Use the terms sex (biological attribute) and gender (shaped by social and cultural circumstances) carefully in order to avoid confusing both terms. Indicate if findings apply to only one sex or gender; describe whether sex and gender were considered in study design whether sex and/or gender was determined based on self-reporting or assigned and methods used. Provide in the source data disaggregated sex and gender data where this information has been collected, and consent has been obtained for sharing of individual-level data; provide overall numbers in this Reporting Summary. Please state if this information has not been collected. Report sex- and gender-based analyses where performed, justify reasons for lack of sex- and gender-based analysis.*
Population characteristics	*Describe the covariate-relevant population characteristics of the human research participants (e.g. age, genotypic information, past and current diagnosis and treatment categories). If you filled out the behavioural & social sciences study design questions and have nothing to add here, write "See above."*
Recruitment	*Describe how participants were recruited. Outline any potential self-selection bias or other biases that may be present and how these are likely to impact results.*
Ethics oversight	*Identify the organization(s) that approved the study protocol.*

Note that full information on the approval of the study protocol must also be provided in the manuscript.

Field-specific reporting

Please select the one below that is the best fit for your research. If you are not sure, read the appropriate sections before making your selection.

☒ Life sciences ☐ Behavioural & social sciences ☐ Ecological, evolutionary & environmental sciences

For a reference copy of the document with all sections, see nature.com/documents/nr-reporting-summary-flat.pdf

Life sciences study design

All studies must disclose on these points even when the disclosure is negative.

Sample size	Power calculations were conducted to determine sample size
Data exclusions	No data were excluded
Replication	All experiments were successfully repeated 2/3 times except for CCR2-/-, CR3CR1-/- animals experiments and monocyte adoptive transfer experiment
Randomization	where appropriate, mice were randomly allocated into the experimental groups. Otherwise, animals were placed into separate groups according to their genotype
Blinding	all experiments were blinded

Reporting for specific materials, systems and methods

We require information from authors about some types of materials, experimental systems and methods used in many studies. Here, indicate whether each material, system or method listed is relevant to your study. If you are not sure if a list item applies to your research, read the appropriate section before selecting a response.

Materials & experimental systems

n/a	Involved in the study	
☐	☒	Antibodies
☒	☐	Eukaryotic cell lines
☒	☐	Palaeontology and archaeology
☐	☒	Animals and other organisms
☒	☐	Clinical data
☒	☐	Dual use research of concern

Methods

n/a	Involved in the study	
☒	☐	ChIP-seq
☐	☒	Flow cytometry
☒	☐	MRI-based neuroimaging

Antibodies

Antibodies used

CD68 antibody (1/250, MCA1957, Biorad, 155083), CD3 staining (1/250, A0452, DAKO, 41246306), 488-AF–anti-rat (1/250, Jackson ImmunoResearch, 712-545-153, 157 033), anti-rabbit IgG (1/250, Jackson ImmunoResearch, 712-545-152, 157 013), AF700-conjugated anti-CD45 (30-F11, 1933401, ThermoFisher Scientific), FITC-conjugated anti-CD11b (M1/70, 1989138, ThermoFisher Scientific), APC-conjugated anti-Ly-6B.2 (clone 7/4, 1804, Biorad), PE-conjugated anti-Ly6G (1A8, 127612, Biolegend), PB-conjugated anti-CD64 (X54-5/7.1, 139309, Biolegend) , APC-conjugated anti-F4/80 (MCA497, 1608, Biorad), PerCP5.5-conjugated anti-MHCII (M5/114.15.2, 107626, BD Biosciences), FITC-conjugated anti-CD4 (clone RM4-5; 4329631, eBioscience), PercpCy5-conjugated anti-CD8 (clone 53-6.7, 553038, BD Pharmingen), APC-conjugated anti-CD3 (clone 17A2, 431133, eBioscience), PE-Cyanine7-conjugated anti-CD11c (cloneN418, 4306332, eBioscience), PE-conjugated anti-NK1.1 (PK136, 557391, BD Pharmingen), VEGF (1:1,000; sc-507, A3113, Santa Cruz Biotechnology), eNOS (1:1000; 610297, 0016148, BD Biosciences), GAPDH (1:10,000, ab8245, GR32355553-2, Abcam)

Validation

All antibodies are used in flow cytometry, immunostaing and western blot have been validated by the respective companies. The validation materials can be found on the companies' website. For flow cytometry, the specificity and sensitivity of each antibody is thoroughly validated. This is done by staining multiple target cells with either single- or multi-color analysis or by other testing approaches. The QC specifications and testing SOPs and gold standard for each product are the developped. The functional performance of each batch of products is strictly QC-tested according to the established QC procedures.

Animals and other research organisms

Policy information about studies involving animals; ARRIVE guidelines recommended for reporting animal research, and Sex and Gender in Research

Laboratory animals

Male C57Bl/6 mice (8 weeks old) were used as experimental animals (Janvier, France). Eight-week-old male CCR2-/-, CR3CR1-/- and their wild-type (WT) C57BL/6 littermates were purchased from the Jackson Laboratories.

Wild animals

this study did not involve wild animals

Reporting on sex

this study did not consider sex

Field-collected samples

this study did not involve field collected samples

Ethics oversight

All animal procedures performed were approved by the institutional animal experimentation and ethics committee (C2EA) at the Institute of Radiation protection and Nuclear Safety (IRSN). Experiments were conducted according to the French veterinary guidelines and those formulated by the European Community for experimental animal use (APAFIS#12903-2018010418086132 v1 and APAFIS #19137-2019021313569870 v1).

Note that full information on the approval of the study protocol must also be provided in the manuscript.

Flow Cytometry

Plots

Confirm that:

☐ The axis labels state the marker and fluorochrome used (e.g. CD4-FITC).

☐ The axis scales are clearly visible. Include numbers along axes only for bottom left plot of group (a 'group' is an analysis of identical markers).

☒ All plots are contour plots with outliers or pseudocolor plots.

☒ A numerical value for number of cells or percentage (with statistics) is provided.

Methodology

Sample preparation

Peripheral blood was drawn via inferior vena cava puncture with heparin solution. Whole blood was lysed after immun¬ofluorescence staining using the BD FACS lysing solution (BD Biosciences), and total blood leukocyte numbers were determined using Kova slides. Bone marrow cells were drawn from femur and filtered through a 40-µm nylon mesh (BD Biosciences). Spleens were collected, gently passed through a 40-µm nylon mesh (BD Biosciences). For both spleno¬cytes and bone marrow–derived cells, the cell suspension was centrifuged at 400g for 10 min at 4°C. Red blood cells were lysed using red blood cell lysing buffer (Sigma-Aldrich) and splenocytes and bone marrow cells were washed with PBS. Non-irradiated and irradiated muscles were collected, minced with fine scissors minced with fine scissors, and gently passed through the Bel-Art Scienceware 12-well tissue disaggregator (Thermo Fisher Scientific). Cells were then filtered through a nylon mesh (40 µm) and centrifuged (10 min, 400g, 4 °C).
Cells isolated from the tissue of interest were incubated in the dark at 4°C for 30 min with the following antibody mix.

Instrument

Canto II, BD Biosciences

Software

DIVA, FlowJo

Cell population abundance	post sort, cell abundance was sufficient for applications
Gating strategy	FSC/SSC gating were used to gate on singlet cells and CD45+ were identified as: Mohi (CD11b+Ly6G–7/4hi), Molo (CD11b +Ly6G–7/4lo), M1-like cells (CD11b+Ly6G-F4/80+CD64+MHCII+) and M2-like cells (CD11b+Ly6G-F4/80+CD64+MHCII-), DC (CD11c+MHCII+), CD4 (CD3+CD4+) and CD8 (CD3+CD4+).

☒ Tick this box to confirm that a figure exemplifying the gating strategy is provided in the Supplementary Information.
